# Defective pro-IL-1β responses in macrophages from aged mice

**DOI:** 10.1186/1742-4933-9-27

**Published:** 2012-12-11

**Authors:** Alejandro Ramirez, Vijay Rathinam, Katherine A Fitzgerald, Douglas T Golenbock, Anuja Mathew

**Affiliations:** 1 Division of Infectious Diseases and Immunology, University of Massachusetts Medical School, S6-862, 55 Lake Avenue North, Worcester, MA, 01655, USA

**Keywords:** Ageing, Macrophages, Aged mice, NLRP3, Inflammasome, IL-1β

## Abstract

**Background:**

Cytokines regulated by the inflammasome pathway have been extensively implicated in various age-related immune pathologies. We set out to elucidate the contribution of the nod-like receptor protein 3 (NLRP3) inflammasome pathway to the previously described deficiencies in IL-1β production by macrophages from aged mice. We examined the production of pro-IL-1β and its conversion into IL-1β as two separate steps and compared these cytokine responses in bone marrow derived macrophages from young (6–8 weeks) and aged (18–24 months) C57BL/6 mice.

**Findings:**

Relative to macrophages from young mice, macrophages from aged mice produced less pro-IL-1β after TLR4 stimulation with LPS. However upon activation of the NLRP3 inflammasome with ATP, macrophages from young and aged mice were able to efficiently convert and secrete intracellular pro-cytokines as functional cytokines.

**Conclusions:**

Lower levels of IL-1β production are a result of slower and lower overall production of pro-IL-1β in macrophages from aged mice.

## Findings

Immunosenescence has been associated with altered levels of pro and anti-inflammatory cytokines such as IL-1β, TNF-α, IL-6, IL-10, TGF-β and Prostaglandin E2 in the blood of aged individuals [1-3]. These aberrant cytokine responses are thought to contribute to the inability of the elderly to mount appropriate immune responses to pathogens, vaccines and self antigen [4]. Macrophages from aged mice have a lower production of cytokines when activated with IFN-γ or LPS [5] and decreased TNF-α and IL-6 production when infected with *Porphyromonas gingivalis*[6] compared to macrophages from young mice. Furthermore bone marrow derived macrophages (BMM) from aged mice secrete less IL-1β compared to BMM from young mice when stimulated with LPS for 6 hours [7]. IL-1β and IL-18 have been associated with a wide variety of age associated diseases including rheumatoid arthritis, multiple sclerosis, atherosclerosis, gout, immunosenescence and greater susceptibility to infectious disease [8-12] thus highlighting the importance of inflammasome regulated cytokines in the aging process [13,14].

The inflammasome is a multiprotein complex that regulates IL-1β and IL-18 secretion by signal recognition via TLRs in combination with NLR or PYHN sensor proteins [15,16]. There are two distinct regulatory steps in the production of active IL-1β. The first step is the production of the biologically inert precursor pro-IL-1β in response to stimulation by a TLR ligand. The second step is the processing of the immature form of the cytokine into its active form by the inflammasome complex. The NLRs are a large family of cytosolic sensors and the best studied one is NLRP3 which forms part of a family of inflammasomes important in detecting many pathogens including influenza virus, *Candida albicans*, *Saccharomyces cerevisiae* and *Staphylococcus aureus*[17]. While previous groups have shown that cells from aged mice produce lower levels of IL-1β, it is unclear whether these defects lie primarily at the priming stage and/or maturation stage of these critical cytokines. The success of immunomodulatory intervention to enhance or dampen aberrant inflammatory cytokine responses in aged individuals will likely depend on identifying and targeting key molecules in inflammatory pathways including the inflammasome.

**Figure 1:**
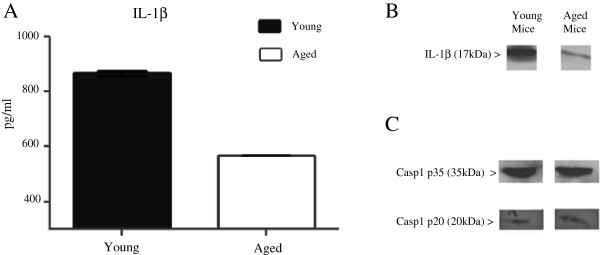
**Macrophages from aged mice produce less functional IL-1β but have similar levels of active Caspase-1.** BMM were primed with LPS (200 ng/ml) for 3 hours and stimulated with ATP (5 mM) for one hour. IL-1β levels in the supernatant of stimulated BMM determined by ELISA (**A**). Data shown are the mean values from triplicate wells +/− SEM. Representative data of at least 3 experiments each performed in triplicate. Immunoblot analysis of the fully processed 17 kDa fragment of IL-1β in supernatants of BMM from young and aged mice stimulated with LPS + ATP (**B**). Immunoblot of active Caspase-1 (p20) subunit and pro Caspase 1 intermediate (p35) in the supernatants from stimulated young and aged BMM (**C**).

**Figure 2:**
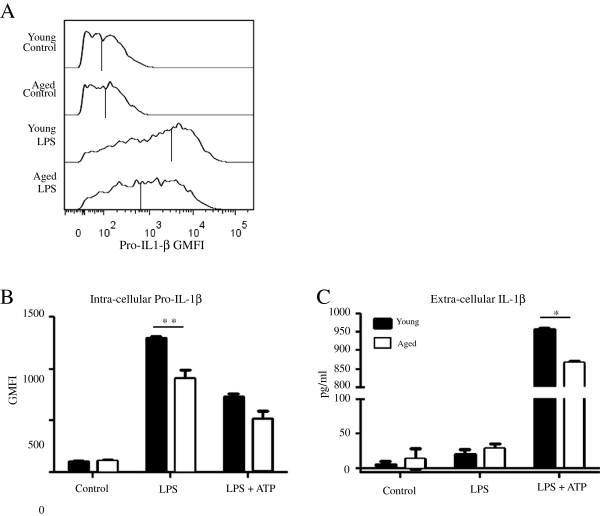
**Efficient processing of pro-IL-1β in BMM from aged mice.** BMM were primed with LPS (200 ng/ml) for 3 hours or primed and stimulated with ATP (5 mM) for one hour (LPS + ATP). Intracellular production of pro-IL-1β as measured by GMFI was assessed in control, LPS and LPS + ATP treated BMM from young and aged mice using the NJTEN3 Ab at 0.05 μg per 10^6^ cells (eBiosciences) (**A**/**B**). Controls were incubated with cell culture medium. ELISA of extracellular active IL-1β in supernatants of control, LPS and LPS + ATP treated BMM from young and aged mice (**C**). Data shown are mean values from triplicate wells +/− SEM. Representative data of at least 3 experiments each performed in triplicate. (* = P <0.05, ** = P < 0.01 Mann-Whitney Test).

**Figure 3:**
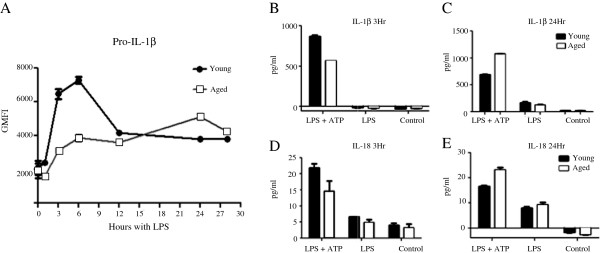
**Age related differences in IL-1β secretion correlate with intracellular pro-IL-1β content.** Time course of intracellular pro-IL-1β production levels in LPS (200 ng/ml) stimulated BMM from young and aged mice using flow cytometry (**A**). ELISA of IL-1β (**B** and **C**) and IL-18 (**D** and **E**) levels in supernatants of young and aged BMM stimulated with LPS (200 ng/ml) for 3 and 24 hours respectively and ATP (5 mM) for 1 hour. Controls were incubated with cell culture medium. Data shown are the mean values from triplicate wells +/− SEM. Representative data of at least 3 experiments each performed in triplicate.

We investigated the contribution of the NLRP3 inflammasome pathway to the previously reported low levels of IL-1β in the aged. Bone marrow cells from young (6–8 weeks) and sex matched aged (18–24 months) mice were differentiated into macrophages using L929 cell conditioned medium containing M-CSF. The resulting cell population expressed F4/80 and CD11b markers with correct myeloid characteristics assessed by flow cytometry (data not shown). BMM from young and aged mice were plated on 24-well plates at a density of 1x10^6^ cells/ml and left to settle overnight at 37 °C. BMM were first primed with LPS (200 ng/ml), a TLR4 agonist, for 3 hours to elicit the production of pro-IL-1β. Following priming, cells were stimulated with the NLRP3 agonist ATP (5 mM) for 1 hour in order to activate assembly of the inflammasome, resulting in pro-caspase activation and ultimately cleavage and extracellular release of IL-1β. Our data indicated that BMM derived from young mice produced significantly more IL-1β in the supernatant as detected by ELISA compared to BMM from aged mice (Figure 1A). Western blots further confirmed a significant increase in levels of fully processed and functional 17 kDa fragment of IL-1β in BMM from young compared to BMM from aged mice. However activation of the NLRP3 inflammasome pathway measured by the presence of active Caspase-1 subunit p20, or the p35 pro-Caspase-1 intermediates were similar in BMM from both young and aged (Figure 1C). The data suggested that defects in BMM from aged mice were likely associated with inadequate production of the proactive cytokine, pro-IL-1β. We next assessed basal levels of pro-IL-1β by using flow cytometry with an antibody which only binds to the pro-from of IL-1β (NJTEN3). We found similar levels of pro-IL-1β measured as geometric mean fluorescence intensity (GMFI) in unstimulated BMM from both young and aged mice (Figure 2A/B). However, after priming with LPS for 3 hours, BMM from young mice had greater intracellular production levels of pro-IL-1β (Figure 2A/B). Following NLRP3 activation with ATP, BMM from both young and aged were able to effectively convert most of the pro-IL-1β content, seen as a reduction in intracellular pro-IL-1β (Figure 2B) and an increase in extracellular levels of active IL-1β (Figure 2C). Our data thus far suggested that the defect in IL-1β production by BMM from older mice occurred at the priming stage. We performed a kinetic analysis of pro-IL-1β production to determine whether the observed differences in BMM from young versus aged were reflective of differences in the kinetics of production or an overall decreased production of pro-IL-1β in BMM from aged mice. Our data indicate decreased pro-IL-1β secretion at early time points (3 and 6 hrs) following LPS priming in BMM from aged (Figure 3A). In BMM from young, pro-IL-1β levels decreased after the 6 hr time point, while levels of pro-IL-1β increased in cells from aged mice up to 24 hrs. To confirm that inflammasome assembly and caspase-1 activity in BMM from aged were similar to BMM from young we activated the NLRP3 pathway at 3 hrs when there was a significantly lower pro-IL-1β levels in BMM from aged mice or 24 hrs where there was a similar intracellular content of pro-IL-1β in BMM from both young and aged after LPS priming. When the NLRP3 complex was activated by ATP at the 24 hr time point, we detected higher levels of IL-1β (Figure 3C) and IL-18 (Figure 3E) in the supernatant of BMM from aged. These results suggest that BMM from aged mice do not have a defect in NLRP3 inflammasome assembly or caspase-1 activity.

Our results taken together indicate that BMM from aged mice may have a significant defect downstream of TLR4 signaling which leads to a reduced output of inflammasome associated cytokines IL-1β and IL-18. BMM from aged mice have a functional IL-1β response but with different kinetics of production as well as a lower overall level of production compared to BMM from young mice. These findings are consistent with the research from other groups which have shown lower secretion of TLR4 dependent cytokines in macrophages from aged mice, which are not dependent upon inflammasome processing, such as TNF-α or IL-6 [18].

Stout-Delgado et al. have recently shown that nigericin, an NLRP3 inducer used in favor of TLR agonists, potentiated IL-1β responses to influenza A virus in aged mice resulting in improved survival and better outcome of infection [19]. Our data indicate that this strategy takes advantage of the fully functional inflammasome response to strong NLRP3 agonists like nigericin or ATP by myeloid cells in aged mice. Our study has implications to understanding immunomodulatory strategies that are needed to boost waning immunity in the elderly [20]. Successful application of these strategies will require controlled stimulation of protective immune responses without exacerbating the release of potentially harmful proinflammatory cytokines.

## Ethical statement

All experiments were performed in accordance with guidelines of the Institutional Animal Care and Use Committee of the University of Massachusetts Medical School and the recommendations in the Guide for the Care and Use of Laboratory Animals (Institute of Laboratory Animal Resources, National Research Council, National Academy of Sciences, 1996).

## Abbreviations

ATP: Adenosine tri-phosphate; BMM: Bone marrow derived macrophages; ELISA: Enzyme linked immune-sorbent assay; GMFI: Geometric mean fluorescence intensity; IFN-γ: Interferon gamma; IL: Interleukin; M-CSF: Macrophage colony stimulating factor; MS: Multiple sclerosis; NLR: Nod like receptor; NLRP3: Nod-like receptor protein 3; LPS: Lipopolysaccharide; SEM: Standard error of the mean; TGF-β: Transforming growth factor beta; TNF-α: Tumor necrosis factor alpha; TLR: Toll like receptor.

## Competing interests

The authors declare that they have no competing interests.

## Authors’ contributions

AR: AB, JY, ES, FG. VR: JY. KAF: FG. DTG: FG. AM: ES, FG. All authors read and approved the final manuscript.
